# SPLICS: a split green fluorescent protein-based contact site sensor for narrow and wide heterotypic organelle juxtaposition

**DOI:** 10.1038/s41418-017-0033-z

**Published:** 2017-12-11

**Authors:** Domenico Cieri, Mattia Vicario, Marta Giacomello, Francesca Vallese, Riccardo Filadi, Tina Wagner, Tullio Pozzan, Paola Pizzo, Luca Scorrano, Marisa Brini, Tito Calì

**Affiliations:** 1 Department of Biomedical Sciences, University of Padova, Padova, Italy; 2 Department of Biology, University of Padova, Padova, Italy; 3 Dulbecco-Telethon Institute, Venetian Institute of Molecular Medicine, Padua, Italy; 4 Venetian Institute of Molecular Medicine, Padua, Italy; 5 Department of Biomedical Sciences, Institute of Neuroscience, Italian National Research Council (CNR), Padua, Italy

## Abstract

Contact sites are discrete areas of organelle proximity that coordinate essential physiological processes across membranes, including Ca^2+^ signaling, lipid biosynthesis, apoptosis, and autophagy. However, tools to easily image inter-organelle proximity over a range of distances in living cells and *in vivo* are lacking. Here we report a split-GFP-based contact site sensor (SPLICS) engineered to fluoresce when organelles are in proximity. Two SPLICS versions efficiently measured narrow (8–10 nm) and wide (40–50 nm) juxtapositions between endoplasmic reticulum and mitochondria, documenting the existence of at least two types of contact sites in human cells. Narrow and wide ER–mitochondria contact sites responded differently to starvation, ER stress, mitochondrial shape modifications, and changes in the levels of modulators of ER–mitochondria juxtaposition. SPLICS detected contact sites in soma and axons of *D. rerio* Rohon Beard (RB) sensory neurons *in* *vivo*, extending its use to analyses of organelle juxtaposition in the whole animal.

## Introduction

In eukaryotic cells, organelles are often found in close proximity, leading to the generation of heterotypic membrane appositions that ensure the coordination of several cellular activities. Indeed, a network of contact sites between membranes of different organelles guarantees their mutual communication by creating microdomains that favor different signaling and metabolic pathways [1,2]. Due to their central role in many fundamental cell processes, the sites of apposition between mitochondria and the endoplasmic reticulum (ER), which range from 10 to 100 nm, are, so far, the best characterized [3–5].

Several approaches are currently available to assess ER–mitochondria contact sites. Electron microscopy (EM) allows to calculate contact site distance, but it is time-consuming. The in situ proximity ligation assay is based on the use of pairs of primary antibodies against proteins on opposing membranes [6]. It is widely used [7–9] but is not devoid of drawbacks: as the EM, it can only be used in fixed cells and is limited by the availability and the specificity of the antibodies.

The use of fluorescent proteins (FPs) selectively targeted to the mitochondrial matrix and the lumen of the ER [10] has been the golden standard to visualize contact sites in living cells for years. However, limited resolution in the distance range below 200 nm, differences in FPs expression levels or alterations in organelle morphology complicated the interpretation of experiments of ER–mitochondria juxtaposition upon ablation of the mitochondria-shaping protein Mitofusin 2 (Mfn2) [11–13].

To overcome these limits, FP-based sensors of proximity were developed: a dimerization-dependent FP (ddGFP) [14] or Venus FP [2,15,16] and a FRET-based probe coupled to a rapamycin-binding module (FEMP) [17]. While these two probes improved the analysis of ER–mitochondria proximity, they also face some limitations: the ddGFP probe is intrinsically not extremely bright [14]; the FRET probe requires equimolar expression of the two moieties [12] and its *in* *vivo* applications are limited by the use of rapamycin, a potent inducer of autophagy [18–20], to maximize juxtaposition and FRET signal. Moreover, both probes cannot be adapted for the investigation of contact sites potentially placed at different distances, because their dynamic range must be characterized each time that the linker is changed. Artificial GFP-based tethers have proved useful to uncover a novel ER–mitochondria tethering complex in yeast, but they cannot been used to monitor changes in the ER–mitochondria contact sites [21]. Therefore, an easy, one-step probe that can dynamically detect ER–mitochondria juxtaposition *in cellulo* and *in vivo* is lacking.

To overcome these limitations, we devised a split-GFP-based contact site sensor (SPLICS) that can be easily adapted to measure ER–mitochondria contact sites over a range of distances as well as other types of hetero and homotypic contact sites. Upon expression in human cells, this one-step imaging technique specifically identifies narrow and wide ER–mitochondria apposition lying in a range of around 10 and 50 nm [5], i.e, that found between mitochondria and smooth or rough ER [22]. The narrow SPLICS can also detect ER–mitochondria contact sites *in* *vivo* in zebrafish sensory neurons. Pharmacological and genetic manipulations indicate that these narrow and wide contact sites respond differentially to ER stress, autophagy, mitophagy, and changes in the levels of modulators of ER–mitochondria juxtaposition.

In conclusion, using SPLICS as a tool to investigate ER–mitochondria contact sites, we unravel their heterogeneity and provide the community with a sensor that can be easily adapted to image other types of heterotypic organelle contact sites in human cells and in whole organisms.

## Results

### Two SPLICS probes for different ranges of ER–mitochondria juxtaposition

**Fig. 1:**
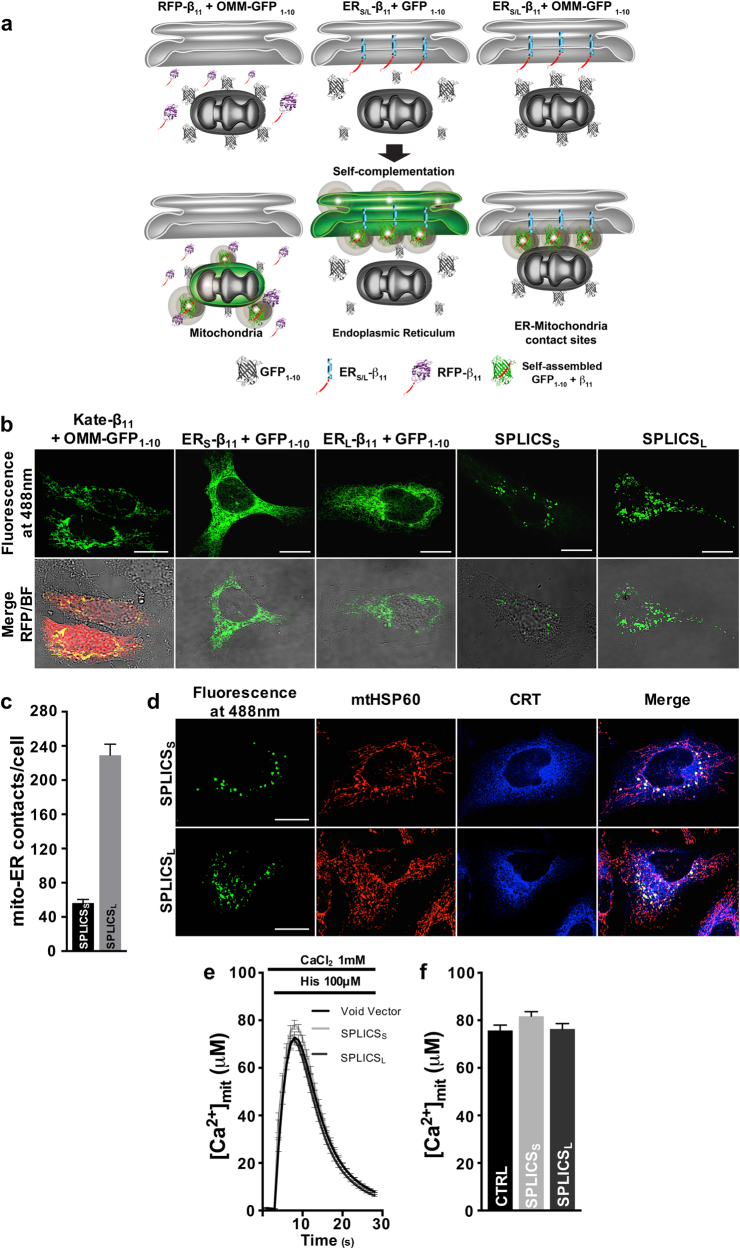
Functional characterization of the SPLICS probes. **a** Cartoon showing the general approach used to design the SPLICS. The mitochondrial network, the ER network, and the ER–mitochondria contact sites are revealed by co-expression of the β_11_-tagged cytosolic RFP (Kate) and the OMM-GFP_1–10_ (left panel), of the ER_S/L_-β_11_ constructs and a cytosolic GFP_1–10_ (middle panel) and of the SPLICS_S/L_ (right panel), respectively. **b** Experimental controls showing the correct targeting of the mitochondrial (OMM-GFP_1–10_) and the ER (ER_S_-β_11_ and ER_L_-β_11_) targeted fragments verified by complementation with Kate-β_11_ and GFP_1–10_, respectively. Co-transfection of HeLa cells with OMM-GFP_1–10_ and both ER_S_-β_11_ or ER_L_-β_11_ induces the appearance of a “dotted” fluorescence. **c** Quantification of ER–mitochondria contacts in HeLa cells. The SPLICS dots were quantified from the 3D rendering of a complete z-stack. Mean ± SEM: SPLICS_S_ 56 ± 4, *n* = 37 cells; SPLICS_L_ 229 ± 12, *n* = 25 cells. **d** Co-localization of SPLICS_S/L_ fluorescence with mitochondria (mtHSP60) and ER (CRT: calreticulin) markers. Representative traces **e** and statistical analysis **f** of mitochondrial Ca^2+^ uptake in HeLa cells transfected with SPLICS_S_ or SPLICS_L_ along with mtAeqmut. Mean ± SEM: Void Vector 75 ± 2, *n* = 65 wells; SPLICS_S_ 77 ± 1, *n* = 54 wells; SPLICS_L_ 71 ± 2, *n* = 54 wells. Scale bar 15 µm. Data shown are the result of 3–5 independent experiments.

To generate a modular fluorescence based sensor of organelle proximity, we decided to capitalize on the ability of two non-fluorescent portions (the GFP_1–10_ moiety and the GFP β-strand 11) of the superfolder GFP variant [23–25] to restore a fully fluorescent GFP upon self-assembly. We reasoned that if we targeted each moiety on one of the juxtaposed membranes, the GFP fluorescence would be restored only when the two portions were close enough. We therefore placed the non-fluorescent GFP_1–10_ moiety on the cytosolic face of the OMM (OMM-GFP_1–10_). To follow short- (≈8–10 nm) and long-range (≈40–50 nm) ER–mitochondria interactions [5], two constructs that differ for the length of the spacer placed between the ER targeting sequence and the β_11_ fragment were created by considering the distance of 0.36 nm between two alpha-carbons in a peptide chain: a ER-Short β_11_ with a 29 aa spacer and a ER-Long β_11_ with a 146 aa spacer (i.e., a maximum of ≈10.4 and 52.5 nm, respectively). These values might clearly be subjected to changes (i.e., reduction) since the amino acid sequences might not always be fully extended. We reasoned that co-expression of ER-Short β_11_ with OMM-GFP_1–10_ (SPLICS_S_) and of ER-Long β_11_ with OMM-GFP_1–10_ (SPLICS_L_) would result in reconstitution of GFP fluorescence (Fig. 1a). Two additional constructs, a β_11_-tagged FP (Kate-β_11_) and an untargeted GFP_1–10,_ were also generated to verify the complementation of the OMM-GFP_1–10_ at the OMM (Fig. 1a, left) and the ER_S/L_-β_11_ at the ER (Fig. 1a, middle), respectively. Expression of SPLICS_S_ and SPLICS_L_ will result in fluorescence emission specifically at the ER–mitochondria interface (Fig. 1a, right).

The different versions of the SPLICS probes were first tested for their correct localization and topology. A clear mitochondrial network appeared in HeLa cells co-expressing OMM-GFP_1–10_ with Kate-β_11_ (Fig. 1b, first panels); similarly, the ER network became fluorescent when ER_S_-β_11_ and ER_L_-β_11_ were co-expressed with a cytosolic non-fluorescent GFP_1–10_ (Fig. 1b, second and third panel couples). Interestingly, when SPLICS_S_ or SPLICS_L_ were expressed in HeLa (Fig. 1b, fourth and fifth panel couples) and in HEK293 cells (Supplementary Figure S1), fluorescent individual foci appeared, likely representing the juxtapositions between ER and mitochondria. At a closer inspection, the SPLICS_S_ and SPLICS_L_ signals retrieved in HeLa cells appeared different for number (see quantification in 3D rendered z-stack images, Fig. 1c).

We therefore verified whether SPLICS really recognized areas of ER–mitochondria juxtaposition. In HeLa cells expressing SPLICS_S/L_, the fluorescent dots co-localized with endogenous markers of mitochondria (mtHSP60) and ER (calreticulin) (Fig. 1d). Noteworthy, the mitochondrial and ER networks were not completely engaged in the formation of the ER–mitochondria contacts reported by the SPLICS (see merge panels of Fig. 1d), suggesting that SPLICS snapshots the juxtaposition at any given moment even when transiently formed. Immuno-EM with anti-GFP antibody revealed that mitochondria and ER membranes in contact with mitochondria were preferentially marked (arrowheads in Supplementary Figure S2). Despite the non-complemented and complemented OMM-GFP_1–10_ cannot be distinguished by the anti-GFP antibody, it is evident that a consistent number of gold nanoparticles are distributed at the ER–mitochondria interface (inset in Supplementary Figure S2).

To gain further insights on the nature of the reconstituted SPLICs, we evaluated their stability by checking whether the number of SPLICS_S/L_ foci could change after 24, 48, and 72 h post transfection. Supplementary Figure S3 shows that the number of SPLICS_S/L_ is stable during the time course. The number of fluorescent reconstituted foci was also unaffected by the expression level of the probes (Supplementary Figure S4), suggesting that bona fide changes in the SPLICS_S/L_ fluorescent foci likely reflect a variation in ER–mitochondria contact sites number rather than differences in the stability/expression levels of the probes. Additionally, the overall morphology of the ER and mitochondria in cells expressing the SPLICS_S/L_ remained grossly unaltered (Supplementary Figure S5).

To exclude that novel and non-physiological contact sites between ER and mitochondria might be artificially induced by SPLICS expression, ER–mitochondria Ca^2+^ transfer and mitochondrial Ca^2+^ uptake were evaluated in HeLa cells expressing SPLICS_S/L_ by aequorin-based measurements. If this was the case, mitochondrial Ca^2+^ transients generated by stimulation with the InsP_3_-linked agonist histamine should be increased in SPLICS-expressing cells [17]; however, they were superimposable to those of control cells (Fig. 1f and quantification in Fig. 1g). Taken together, these results indicate that SPLICS retains the ability to self-associate only in specific areas where the two organelles are found within the distance imposed by the linker region and that it does not artificially increase tethering and Ca^2+^ transfer between ER and mitochondria.

### Modulation of short- and long-range ER–mitochondria interfaces during ER stress and autophagy

**Fig. 2:**
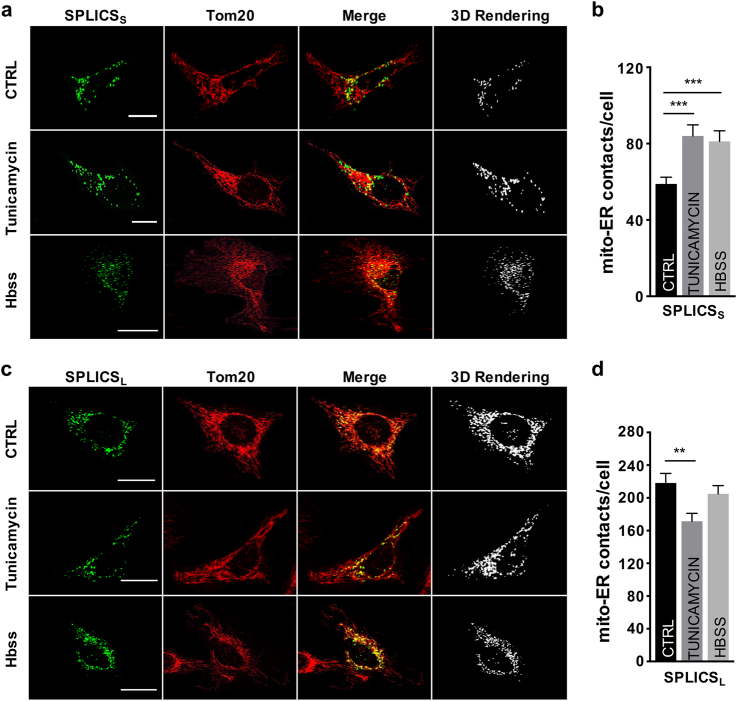
Effect of Tunicamycin and Hbss treatment on ER–mitochondria contacts. Immunofluorescence against mitochondria (Tom20, red) is shown in the panels on the middle. The green channel is the merge of several planes. Scale bar 20 µm. **a** Representative confocal pictures of HeLa cells expressing the SPLICS_S_ probe. **b** Quantification of SPLICS_S_ contacts by 3D rendering of complete z-stacks. Mean ± SEM: Ctrl 58 ± 3, *n* = 32 cells; Tunicamycin 84 ± 5, *n* = 33 cells; Hbss 81 ± 5, *n* = 25 cells. **c** Representative confocal pictures of HeLa cells expressing the SPLICS_L_ probe. **d** Quantification of SPLICS_L_ contacts by 3D rendering of complete z-stacks. Mean ± SEM: Ctrl 218 ± 11, *n* = 27 cells; Tunicamycin 171 ± 9, *n* = 33 cells; Hbss 204 ± 10, *n* = 23 cells. Data shown are the result of three independent experiments. **p ≤ 0.01, ***p ≤ 0.001.

We next wished to address if SPLICS could respond to pathophysiological conditions known to affect the extent of ER–mitochondria contacts. We therefore measured short- and long-range ER–mitochondria interactions in conditions where increased ER–mitochondria coupling was reported, such as ER stress and induction of autophagy [5,26,27]. In HeLa cells treated with the ER stress inducer tunicamycin, or starved, the number of short-range ER–mitochondria contact sites measured by SPLICS_S_ were increased (Figs. 2a,b), in agreement with previous results [5,26,27]. The picture in the case of long-range ER–mitochondria interactions measured by the SPLICS_L_ was more complex: while tunicamycin significantly decreased the number of SPLICS dots, starvation did not induce any significant change (Figs. 2c,d). Altogether, these results indicate that short and long ER–mitochondria interactions are differentially modulated in response to different stimuli and suggest that the heterogeneity between the two types of contact sites reflects their involvement in specialized cellular pathways.

### Short- and long-range ER–mitochondria interactions are differentially modulated by mitochondrial morphology

**Fig. 3:**
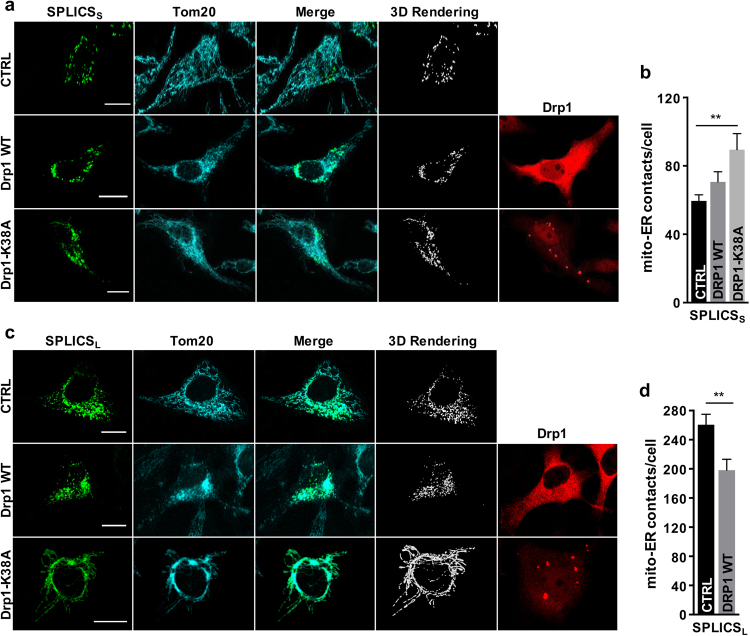
Effects of Drp1 overexpression on ER–mitochondria contacts. Immunofluorescence against mitochondria (Tom20, cyan) and Drp1 (red) is shown in the corresponding panels. The green channel is the merge of several planes. Scale bar 20 µm. **a** Representative confocal pictures of HeLa cells expressing the SPLICS_S_ probe. **b** Quantification of SPLICS_S_ contacts by 3D rendering of complete z-stacks. Mean ± SEM: Ctrl 59 ± 3, *n* = 79 cells; Drp1 WT 70 ± 5, *n* = 32 cells; Drp1-K38A 89 ± 9, *n* = 28 cells. **c** Representative confocal pictures of HeLa cells expressing the SPLICS_L_ probe. **d** Quantification of SPLICS_L_ contacts by 3D rendering of complete z-stacks. Mean ± SEM: Ctrl 260 ± 14, *n* = 24 cells; Drp1 WT 198 ± 14, *n* = 24 cells. Data shown are the result of 3–4 independent experiments. **p ≤ 0.01.

During starvation, inhibition of the mitochondrial fission GTPase Dynamin-related protein 1 (Drp1) results in mitochondrial elongation, increasing energy conversion and sparing mitochondria from autophagosomal degradation [28,29]. We therefore wished to verify short and long ER–mitochondria interactions upon Drp1-driven mitochondrial shape changes. We expressed wt or a dominant-negative mutant form of Drp1 (Drp1-K38A) to induce mitochondrial fragmentation or elongation and measured the occurrence of short- and long-range ER–mitochondria juxtaposition with SPLICS. Mitochondrial fragmentation induced by wt Drp1 expression did not change the number of short-range ER–mitochondria interactions (Figs. 3a and b, compare top panels vs. middle panels), in agreement with previous data [30]. Conversely, mitochondrial elongation induced by dominant-negative Drp1 expression resulted in a significant increase in the short-range ER–mitochondria contacts detected by SPLICS_S_ (Fig. 3a, top panels vs. lower panel, and Fig. 3b). The SPLICS_L_ measured a significant reduction in the number of wide ER–mitochondria interactions in cells expressing wt Drp1 (Fig. 3c top panels vs middle panels, and Fig. 3d). Interestingly, forced mitochondrial elongation induced by Drp1-K38A expression resulted in the labeling of the whole surface of mitochondria by SPLICS_L_ fluorescence, suggesting a complete engagement of the mitochondrial network with the ER (Fig. 3c, top panels vs. bottom panels). Due to the filamentous nature of the observed SPLICS_L_ staining, the number of ER–mitochondria contacts/cell under this condition could not be reliably quantified; nevertheless, the GFP signal occupied almost completely (about 85%) the mitochondrial surface as measured by Tom20 staining (Supplementary Figure S6). Altogether, these results suggest that unopposed mitochondrial fusion is paralleled by an enhancement of the ER–mitochondria interface that may ensure the supply of lipids required for the sustained mitochondrial morphological changes [26,28,29,31].

### Short- and long-range ER–mitochondria contacts respond differentially to Mfn2 silencing and presenilin 2 mutant expression

**Fig. 4:**
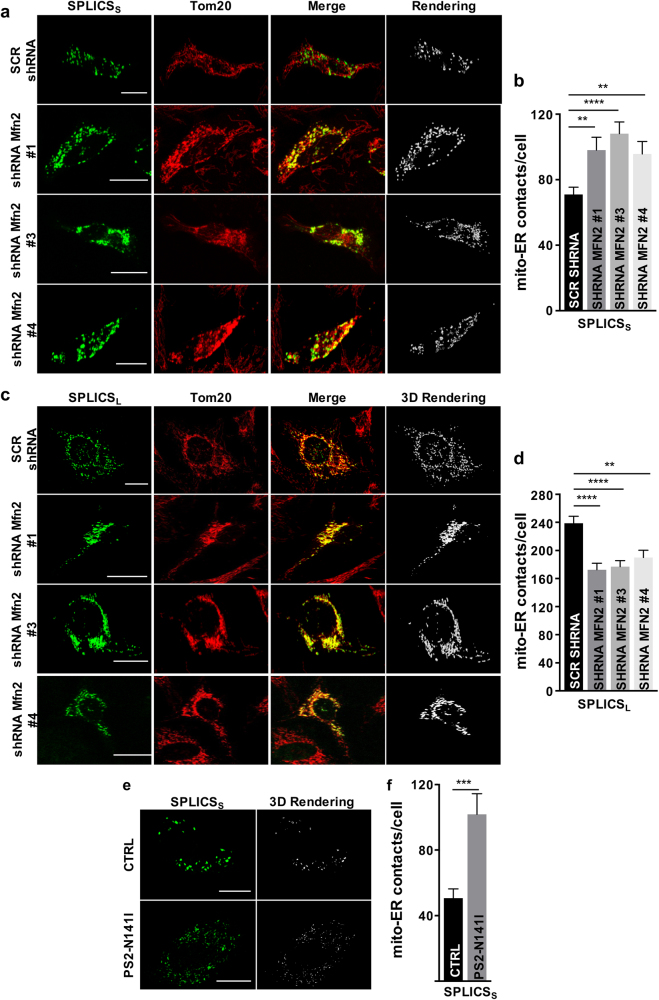
Effect of Mfn2 knockdown and mutant PS2 on ER–mitochondria interface. Immunofluorescence against mitochondria (Tom20, red) is shown in the panels on the middle. The green channel is the merge of several planes. Scale bar 20 µm. **a** Representative confocal pictures of HeLa cells expressing the SPLICS_S_ probe. **b** Quantification of SPLICS_S_ contacts by 3D rendering of complete z-stacks. Mean ± SEM: SCR shRNA 70 ± 4, *n* = 76 cells; shRNA Mfn2 #1 98 ± 7, *n* = 26 cells; shRNA Mfn2 #3 108 ± 7, *n* = 28 cells; shRNA Mfn2 #4: 95 ± 7, *n* = 23 cells. **c** Representative confocal pictures of HeLa cells expressing the SPLICS_L_ probe. **d** Quantification of SPLICS_L_ contacts by 3D rendering of complete z-stacks. Mean ± SEM: SCR shRNA 238 ± 10, *n* = 30 cells; shRNA Mfn2 #1 172 ± 9, *n* = 27 cells; shRNA Mfn2 #3 176 ± 8, *n* = 29 cells; shRNA Mfn2 #4 190 ± 10, *n* = 27 cells. **e** Representative confocal pictures of human fibroblasts from a patient with the N141I mutation in PS2 (bottom panel) and an age-matched control (upper panel) expressing the SPLICS_S_ probe. The green channel is the merge of several planes. Scale bar 20 µm. **f** Quantification of ER–mitochondria short contacts by 3D rendering of complete z-stacks. Mean ± SEM: CTRL 50 ± 5, *n* = 20 cells; PS2-N14I: 101 ± 12, *n* = 21 cells. Data shown are the result of 2–5 independent experiments. **p ≤ 0.01, ***p ≤ 0.001, ****p ≤ 0.0001.

We next wished to verify if SPLICS responded to genetic modulation of the ER/mitochondria interaction. To this end, we decided to monitor SPLICS_S/L_ behavior following ablation of Mitofusin 2 (Mfn2), a pro-fusion mitochondria-shaping protein originally identified as a tether between the two organelles [11]. However, whether Mfn2 tethers [12,14,32–35] or separates [13,22,36–38] ER and mitochondria is still a matter of debate. We reasoned that SPLICS_S/L_ might contribute to clarify the issue by providing an estimate of the contact sites over different ranges of interaction. Acute downregulation of Mfn2 by shRNA in HeLa cells by three independent shRNA (Supplementary Figure S7) increased by ≈40% the number of SPLICS_S_ foci (Figs. 4a,b). Conversely, under the same conditions of Mfn2 downregulation the SPLICS_L_ detected a significant decrease by ≈30% in the number of ER–mitochondria interactions (Figs. 4c,d). Altogether the short- and long-range SPLICS probes not only respond to changes in known modulators of ER–mitochondria tethering, but they might also prove useful to shed light on the observed discrepancies on the role of Mfn2 at the ER–mitochondria interface.

Mfn2 and the Familial Alzheimer’s Disease (FAD)-related protein Presenilin-2 (PS2) have been reported to act in a common route to tune the ER–mitochondria interface [38,39]. We measured short-range ER–mitochondria interactions in human fibroblasts from an FAD-patient carrying the PS2-N141I mutation, previously shown to enhance ER–mitochondria coupling in an Mfn2-dependent manner [38], and a healthy sex- and age-matched control. The SPLICS_S_ signal was more than doubled in human FAD-PS2 fibroblasts compared to controls, thus confirming that endogenous FAD-PS2 increases ER–mitochondria coupling, as already reported, and proving that SPLICS represents a useful tool also in patient-derived samples (Figs. 4e,f). Lastly, we tested SPLICS_S/L_ with an additional well-established tethering machinery, i.e., the VAPB/PTPIP51 complex. Interestingly, we detected an increase in the SPLICS_S_ number, in agreement with previous data [7,8,40] (Supplementary Figure S8). The long-range interactions monitored by SPLICS_L_ were instead decreased (Supplementary Figure S8): this finding certainly deserves additional experiments but again, it might indicate that ER–mitochondria tethering can be heterogeneous and tightly modulated.

### Long- and short-range ER mitochondria contacts reduction during Parkin-mediated mitophagy

**Fig. 5:**
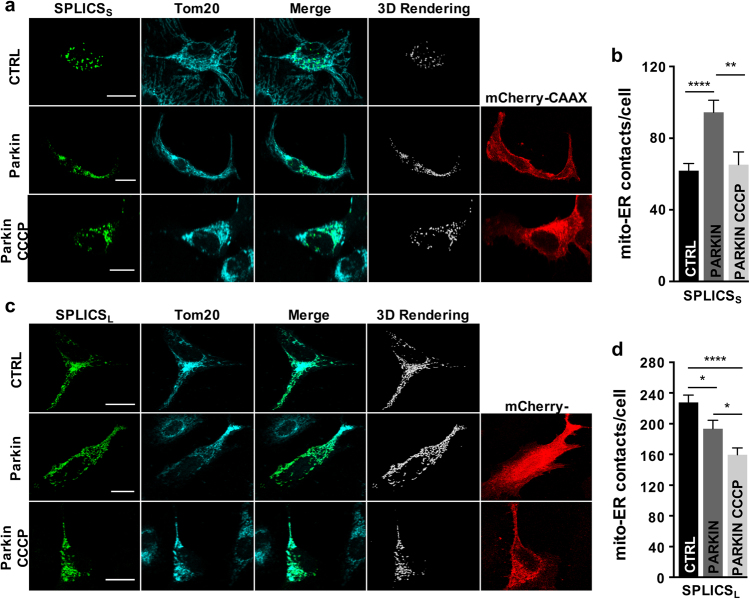
Effects of Parkin on ER–mitochondria contacts. Immunofluorescence against mitochondria (Tom20, cyan) is shown in the panels on the middle. The green channel is the merge of several planes. Scale bar 20 µm. **a** Representative confocal pictures of HeLa cells expressing the SPLICS_S_ probe along with Parkin-2A-mCherry-CAAX (bottom panels). **b** Quantification of SPLICS_S_ contacts by 3D rendering of complete z-stacks. Mean ± SEM: Ctrl 61 ± 3, *n* = 57 cells; Parkin 94 ± 6, *n* = 33 cells; Parkin CCCP 65 ± 7, *n* = 26 cells. **c** Representative confocal pictures of HeLa cells expressing the SPLICS_L_ probe along with Parkin-2A-mCherry-CAAX (bottom panels). **d** Quantification of the SPLICS_L_ contacts by 3D rendering of complete z-stacks. Mean ± SEM: CTRL 227 ± 9, *n* = 44 cells; Parkin 193 ± 11, *n* = 32 cells; Parkin CCCP 159 ± 8, *n* = 22 cells. Data shown are the result of 3–8 independent experiments. *p ≤ 0.05, **p ≤ 0.01, ****p ≤ 0.0001.

Comforted by the ability of SPLICS_S/L_ to provide insights under pharmacological and genetic manipulation of the ER–mitochondria interface, we decided to detect changes in ER–mitochondria tethering during Parkin-mediated mitophagy. In mammalian cells, dysfunctional mitochondria recruit the E3 ubiquitin ligase Parkin to the OMM through PINK1 kinase activity, resulting in the recruitment and activation of the autophagy machinery [41]. Parkin has been shown to act as a positive modulator of ER and mitochondria coupling in HeLa cells by organelle-targeted FPs, and in nigral neurons by transmission EM analysis [42,43]. Nevertheless, increased ER–mitochondria juxtaposition in patient-derived fibroblasts and in *PARK2* knockout MEFs [44] was also reported. Thus, the exact function of Parkin at the ER–mitochondria interface under basal conditions and upon mitophagy is unclear. We generated a bicistronic vector in which Parkin was cloned upstream of a self-cleaving viral 2A peptide (P2A) [45] followed by a plasma membrane-targeted RFP (mCherry-CAAX) to track Parkin-positive cells (Supplementary Figure S9). This construct was co-expressed along with SPLICS_S/L_ in HeLa cells where Parkin is absent or weakly expressed [46,47]. Parkin overexpression increased SPLICS_S_ number (Figs. 5a,b), in agreement with our previous data [42]. Conversely, Parkin overexpression reduced the SPLICS_L_ foci (Figs. 5c,d). Treatment with CCCP reduced the number of fluorescent foci measured using both the SPLICS probes, suggesting that activation of PINK1/Parkin-mediated mitophagy loosens all types of ER–mitochondria contacts.

### SPLICS visualizes ER–mitochondria interactions in living zebrafish neurons

**Fig. 6:**
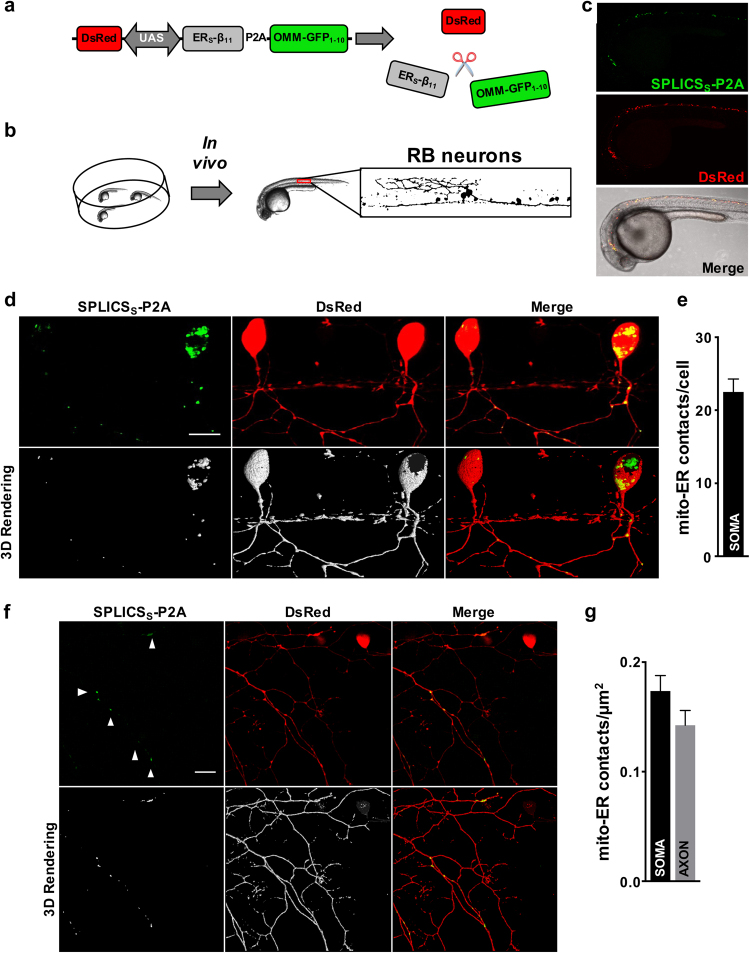
Expression of the SPLICS_S_ probe in living zebrafish embryos. **a** Schematic depiction of the bidirectional construct that allows detection of DsRed and SPLICS_S_-P2A in a Gal4-dependent manner. The 2A peptide guarantees the generation of an equimolar amount of the two spGFP fragments. **b** Experimental setting used to image ER–mitochondria contacts in zebrafish embryos: a schematic drawing of RB neurons is shown on the right. **c** Representative image of a 1 dpf s1102t:GAL4 embryo injected with the pT2-DsRed-UAS-SPLICS_S_-P2A construct. **d** Live imaging of short ER–mitochondria contacts in RB neurons of s1102t:GAL4 zebrafish embryos. The picture is the merge of several planes. The 3D rendering of the z-stack is shown on the right. Scale bar: 15 µm. **e** Quantification of SPLICS_S_ contacts in the cell body of RB neurons by 3D rendering of complete z-stacks. Mean ± SEM: 22 ± 1, *n* = 28 cells from 11 fish. **f** Live imaging of SPLICS_S_ contacts in the axons of RB neurons. The picture is the merge of several planes. The 3D rendering of the complete z-stack is shown on the right. Scale bar: 15 µm. **g** Quantification of the density of SPLICS_S_ contacts in the cell body and the axons of RB neurons. Mean ± SEM: RB soma: 0.17 ± 0.01, *n* = 28 cells from 11 fish; RB axon: 0.14 ± 0.01, *n* = 20 cells from 6 fish. Data shown are the result of two independent experiments.

We finally wished to test if SPLICS can measure ER–mitochondria tethering in an *in vivo* setting. Imaging of subcellular structures in living animals, and even more in neuronal axons, is limited by the thickness and anatomical accessibility of tissues. *In vivo* detection of organelle contact sites is still a major challenge because of their dynamic nature and the lack of appropriate tools. To verify if SPLICS could overcome these hurdles, we expressed the new probes in *D. rerio*, specifically in Rohon-Beard (RB) sensory neurons. The correct targeting of the OMM-GFP_1–10_ and the ER_S_-β_11_ constructs was first verified after mosaic expression in *D. rerio* embryos. The OMM-GFP_1–10_ signal reconstituted by complementation with a β_11_-tagged cytosolic protein (DJ-1-β_11_) fully overlapped with a mitochondrial targeted RFP (pTagRFP-mito). Analogously, injection of ER_S_-β_11_ and a cytosolic GFP_1–10_ resulted in fluorescence emission that co-localized with an ER marker (pDsRed2-ER) (Supplementary Figure S10), thus demonstrating that the SPLICS fragments are properly expressed, targeted and self-assembled in living zebrafish embryos. To allow tissue specific as well as equimolar expression of SPLICS, we generated an expression vector where OMM-GFP_1–10_ and ER_S_-β_11_ are linked by a P2A peptide (SPLICS_S_-P2A), an approach suitable also in zebrafish [48]. SPLICS_S_-P2A was placed under the control of a bidirectional UAS promoter together with a cytosolic DsRed (pT2-DsRed-UAS-SPLICS_S_-P2A) to allow GAL4-driven expression of the UAS promoter (Fig. 6a). The pT2-DsRed-UAS-SPLICS_S_-P2A vector was then microinjected in the zebrafish s1102t:GAL4 transgenic line where GAL4 expression is restricted to RB neurons (Fig. 6b), yielding simultaneous, tissue specific expression of DsRed and SPLICS_S_ (Fig. 6c). By imaging the DsRed-positive neurons, we noticed the occurrence of short-range ER mitochondria contacts in both cell body and axons (Figs. 6d–g). We retrieved several SPLICS_S_ contacts in the soma of RB neurons; their frequency was comparable to that observed in cultured cells. ER–mitochondria contact sites were also retrieved in RB axons and enriched at axonal varicosities and branching points, possibly representing axon zones with specialized functions where ER–mitochondria crosstalk is important to propagate and regulate Ca^2+^ signals [49–51] (arrowheads in Fig. 6f). The number of short ER–mitochondria interactions was comparable in soma and axons (Fig. 6g), suggesting that these juxtapositions are regulated by similar mechanisms in the two portions of the neuron.

## Discussion

Here we report a one-step, split-GFP-based method to assess narrow and wide ER–mitochondria contact sites and their modulation and demonstrate its suitability to monitor by confocal microscopy inter-organelle interactions in human cells and *in vivo* in zebrafish RB neurons.

The SPLICS is versatile and sensitive and reveals short and long-range ER–mitochondria interactions and their changes upon pharmacological and genetic manipulations.

An advantage of SPLICS over the other probes relies not only on its unnerving modularity, but also on the high brightness, stability and a high threshold over background. The first feature was exploited here to generate two versions of SPLICS, one engineered to detect narrow (≈8–10 nm) and a second to image wide (≈40–50 nm) ER–mitochondria interactions [5,13]. Although the low dissociation rate of the GFP fragments [23] could, upon reconstitution, make their association poorly reversible (implying that any reduction observed with SPLICS_S/L_ may not reflect a dynamic decrease during time). Even if the observation of transient interactions between the ER and motile mitochondria could be limited by the time required to achieve full reconstitution of the SPLICS_S/L_, we were able to provide important insights in the biology of this interface.

SPLICS can be also adapted to monitor other types of heterotypic organelle contact sites, e.g., ER and plasma membrane (PM), mitochondria and PM, or mitochondria and endosomes/lysosomes, creating a palette of SPLICS to image inter-organelle interactions.

The physiological significance of long-range ER–mitochondria contacts has not been completely defined; nevertheless, the comparison of the SPLICS_S/L_ signals under different pathophysiological conditions indicates that ER–mitochondria tethering is heterogeneous and tightly modulated. Both ER stress and starvation increased SPLICS_S_ foci while SPLICS_L_ dots were decreased in number under ER stress, suggesting a spatial and functional specialization of different ER–mitochondria contact sites [27]. Changes in mitochondrial shape also affected the ER–mitochondria interface differently: Drp1 overexpression reduced SPLICS_L_ interactions, whereas forced mitochondrial elongation increased both SPLICS_S/L_ foci number. Fragmentation concomitantly ensures basal Ca^2+^-dependent homeostatic mitochondrial functions and protects from stress responses involving ER–mitochondria Ca^2+^ crosstalk [30]. Indeed, potentiation of the ER–mitochondria interface under conditions of Drp1-dependent fragmentation can lead to mitochondrial Ca^2+^ overload and cell death. The reduction in SPLICS_L_ number is probably due to the reduction of the interface available for additional contacts and it could be relevant in the Ca^2+^-dependent stress responses. Of note, Drp1-dependent mitochondrial fission facilitates mitophagy whereas mitochondrial elongation inhibits mitochondrial autophagy [52]. Thus, it is tempting to speculate that mitochondria are protected from mitophagy when mitochondrial fusion is forced due to increased ER–mitochondria contacts; conversely, mitochondrial fission is accompanied by a reduction in SPLICS_L_ signal to favor the engulfment of mitochondria by the autophagosome.

The SPLICS described here can also help to understand the role of Mfn2 in ER–mitochondria juxtaposition: we observed that Mfn2 silencing led to an increase in SPLICS_S_ and a decrease in SPLICS_L_ dots. Similar findings were observed upon the overexpression of the well-established tethering machinery VAPB/PTPIP51. Future work might therefore capitalize on spectral variant of the SPLICS_S/L_ probes to verify if the wide interactions comprise the narrow ones, or if they occur on different areas of the organelles.

CCCP treatment in HeLa cells expressing Parkin caused a marked reduction in both SPLICS_S_ and SPLICS_L_ signal, supporting a model whereby mitochondria and ER separate during mitophagy. Under basal conditions, overexpression of Parkin conversely increases ER–mitochondria tethering in several cellular models [42,43], suggesting a housekeeping function at this interface, whereas massive Parkin activation and mitophagy induction mirrors a condition of degradation/deactivation of a handful of molecules involved in ER–mitochondria juxtaposition (e.g., VDAC, Mfn2, TOM20) [53] with consequent ER–mitochondria separation.

Despite the efforts in the analysis of the ER–mitochondria contact sites, their properties in neurons and, most importantly, in living vertebrates are still poorly characterized due to the lack of appropriate methods suitable for *in vivo* studies. We optimized the SPLICS for *in vivo* expression by generating the pT2-DsRed-UAS-SPLICS_S_-2A construct. SPLICS could report ER–mitochondria contacts in cell body and axons of RB neurons in living zebrafish embryos. ER–mitochondria contacts in the axons often localized at sites of branching, a process sustained by mitochondrial ATP [54] and Ca^2+^ release from internal stores [55], thus suggesting that ER–mitochondria interplay may have a role in this process. SPLICS is a versatile tool to visualize ER–mitochondria contacts *in vivo* both in physiological and pathological conditions and it will be useful to explore how disease-related genes affect neuronal function and survival through the modulation of the ER–mitochondria interface.

## Materials and methods

### Cell Lines

HeLa and HEK293 cells (ATCC) were grown in a 5% CO_2_ atmosphere in Dulbecco’s modified Eagle’s medium high glucose (DMEM; Euroclone), supplemented with 10% fetal bovine serum (Gibco), 100 U/ml penicillin and 100 mg/ml streptomycin. Where indicated, the cells were treated 48 h after transfection with 10 µM CCCP (Sigma-Aldrich), Hbss (Thermo Fisher) or 10 µg/ml Tunicamycin (Sigma-Aldrich) for 4 h at 37 °C, in a 5% CO_2_ atmosphere. Mock cells were maintained in growth medium, which was changed simultaneously with the beginning of treatments. Female FAD-PS2-N141I fibroblasts (Coriell Institute, AG09908) and age/sex-matched control fibroblasts (Coriell Institute, AG08269) were grown in DMEM containing FCS (15%), l-glutamine (2 mM), penicillin (100 U/ml, Euroclone), and streptomycin (100 µg/ml, Euroclone).

### Zebrafish husbandry and transgenic lines

All animal experiments were conducted as previously reported [56]. Adult fish were maintained and raised in 5 l tanks with freshwater at 28 °C with a 12 h light/12 h dark cycle. Zebrafish embryos were obtained from spontaneous spawnings. To obtain fish selectively expressing Gal4 in Rohon-Beard neurons (s1102t:GAL4), Et(E1b:GAL4-VP16)s1102t; Tg(UAS-E1b:Kaede)s1999t fish (ZIRC, ZL1384) were outcrossed with wild-type (wt) fish [57] and the fluorescent offspring was discarded. The remaining fish were genotyped from fin clips with primers specific for GAL4. To perform experiments, both wt and s1102t:GAL4 fish were used. All experiments were conducted on 24 h post fertilization (hpf) embryos.

### Transfection

Twelve hours before transfection, HeLa cells were seeded onto 13 mm glass coverslips and allowed to grow to 50% confluence. Cells were transfected by calcium phosphate [58]. For co-transfection, the two SPLICS ER and mitochondrial fragments were in a 1.5:2 ratio with the overexpressed protein of interest. Human fibroblasts were electroporated by using a NeonTM transfection system (Life Technologies), according to the manufacturer's instruction.

### Cloning and fusion plasmid construction

Humanized GFP_1–10_ (GFP_1–10(h)_) was PCR amplified from the GPI-GFP_1–10(h)_ kindly provided by Prof. Fabien Pinaud, Department of Biological Sciences and Chemistry, University of Southern California [59], to insert the Tom20 N33 targeting sequence [60] by using the OMM-GFP_1–10_ For. (TCGAATTCATGGTGGGCCGGAACAGCGCCATCGC CGCGGGCGTGTGCGGTGCCCTCTTCATAGGGTACT GCATCTACTTTGACCGCAAAAGGCGGAGTGACCC CAACTCCAAAGGAGAAG) and Rev. (AC TTCTCACT CGAGTTATGTTCCTTTTTCATTTGGATCTTTGCTCA GG) primers and inserted into the vector pcDNA3 between the *Eco*RV and *Xho*I restriction sites. The chimeric sequences composed by the minimal *Sac*1 ER targeting sequence and the *Sac*1 ER targeting sequence containing additional 267 bp of the helix-FRB fragment (derived from the pEGFP-C3/CFP-HA-FRB-helix-ER plasmid [5]) (ER_L_-β_11_) were fused with the β_11_ tag to generate the ER_s_-β_11_ and the ER_L_-β_11_ constructs, respectively. The constructs were chemically synthetized (Thermo Fisher) by using the *Eco*RI/*Not*I and the *Eco*RV/*Xho*I restriction sites of pCDNA3.1(+), respectively. The sequence encoding for β_11_, flanked by different multi cloning sites, has been inserted into the commercial vectors pCDNA6.5/V5-DEST (Invitrogen), in order to obtain a backbone that could easily allow fusion of the β_11_ fragment with the protein of interest. Kate was amplified with the Kate For. (AAAAAAGCTTATGGTGAGCGAGC) and the Kate Rev. (TTTTTG GTACCTCATCTGTGCC) primers using as a template mKate2-pcDNA3.1 (pEVROGEN) and inserted in pDEST-β_11_ to generate Kate β_11_. To generate a vector simultaneously expressing (through a 2A peptide) Parkin and membrane mCherry (CAAX-mCherry), the coding sequence of Parkin [42] was amplified with the primers Parkin For. (ACGCGGATCCGCCACCATGATAGTGTT TGTCAGGTT) and Rev. (TTCCCCCGGGCA CGTCGAA CCAGTGGTCC). The PCR products were purified using the GenElute Gel Extraction Kit (Sigma), digested with *Bam*HI and *Sma*I and then ligated into the pSYC-187 vector (Addgene) digested with the same restriction enzymes. The constructs codifying for Drp1 WT and K38A [30,61,62] and pmTurquoise2-ER were kindly donated by Diego De Stefani (Department of Biomedical Sciences, University of Padova). The pCIneo-PTPIP51-HA and the pCIneo-VAPB-Myc were kindly provided by Professor Christopher C.J. Miller (Department of Neuroscience and Department of Basic and Clinical Neurosciences, King’s College London). Where indicated, mitochondria are labeled with a pTagRFP-mito construct (Evrogen). To knockdown Mfn2, three different shRNAs against Mfn2 were used (SureSilencing shRNA Plasmid, Hygromycin Gene: Mfn2; Refseq Accession #:NM_014874; Clone #1: AGAGGCGGTTCGACTCATCAT; Clone #3: TGATGTG GCCCAACTCTAAGT; Clone #4: CCAGTAGTCCTCAA GGTTTAT; Scramble: GGAATCTCATTCGATGCATA C). The aminoacid sequences of the ER anchored probes are: MRDHMVLHEYVNAAGITGGDGGSGGGSKLRVF LALPIIMVVAFSMCIICLLMAGDTWTETLAYVLFWG VASIGTFFIILYNGKDFVDAPRLVQKEKID (the Short) and MRDHMVLHEYVNAAGITGGDGGSGGGSKLMW HEGLEEASRLYF GERNVKGMFEVLEPLHAMMERGP QTLKETSFNQAYGRDLMEAQEWCRKYMKSGNVKD LTQAWDLYYHVFRRISKQGSEAAAREAAARGGASG AGAGAGAILNSRVFLALPIIMVVAFSMCIICLLMAGD TWTETLAYVLFWGVASIGTFFIILYNGKDFVDAPRLV QKEKID (the Long).

### Zebrafish constructs

To properly express SPLICS in zebrafish, the ER_S_-β_11_ and the OMM-GFP_1–10_ coding sequences were respectively cloned upstream and downstream of a viral 2A peptide sequence (pSYC-181, Addgene), previously reported to be cleaved within the cell to generate an equimolar amount of the two genes [48]. The fragment encoding for the OMM-GFP_1–10_ was excised from pcDNA3 with *Eco*RI and *Xho*I and ligated into the pSYC-181 vector digested with the same enzymes. Since these sites cloned the OMM-GFP_1–10_ fragment out of frame, we mutagenized the resulting vector in order to re-establish the correct frame, using the primer OMM-GFP_1–10_ mut. (CCCTGGACCTAGATCTGAATTC ATGGTGGGCC). ER_S_ was amplified from pcDNA3 using the primers ER_S_-β_11_ 2A For. (ACGCGGATCCGCCACCATGCGGGACCACATGGTG C) and Rev. (TTCCCCCG GGGTCGATCTTCTCTTT), which introduce, respectively, a *Bam*HI-Kozak sequence and a *Sma*I site at the 5′ and 3′ ends of the ER_S_ coding sequence. The PCR product was purified, digested with both *Bam*HI and *Sma*I and then ligated into the pSYC-181-OMM-GFP_1–10_ vector, previously digested with the same restriction enzymes. We refer to this plasmid as pSYC-SPLICS_S_-P2A. To selectively express the SPLICS_S_-P2A in zebrafish neurons, we exploited the Gateway technology to generate the pT2-DsRed-UAS-SPLICS_S_-P2A vector. Briefly, the fragment encoding for the SPLICS_S_-P2A was excised from the pSYC-SPLICS_S_-P2A vector with *Bam*HI and *Xba*I and cloned into the *Bam*HI-*Xba*I sites of the pME-MCS vector (Tol2 kit). The resulting pME-SPLICS_S_-P2A was recombined through the LR reaction with the pT2d­DESTpADs Red.T4E1bUASE1bGW­R1­R2pA vector (kindly donated by Christian Haas, Ludwig-Maximilians University Munich). The resulting pT2-DsRed-UAS-SPLICS_S_-P2A plasmid was injected into 1–2 cells stage s1102t:GAL4 embryos. To label mitochondria and the endoplasmic reticulum, the pTagRFP-mito (Evrogen) and the pDsRed2-ER (Clontech) plasmids were, respectively, injected in WT eggs. For injections, all plasmids were diluted in Danieau solution (58 mM NaCl, 0.7 mM KCl, 0.4 mM MgSO_4_, 0.6 mM Ca(NO_3_)_2_, 5 mM HEPES pH 7.6) and 0.5% phenol red.

### Immunocytochemistry

To image cells over-expressing a protein of interest, transfected cells plated on 13 mm glass coverslips were fixed 48–72 h post transfection with 3.7% formaldehyde in phosphate-buffered saline (PBS; 140 mM NaCl, 2 mM KCl, 1.5 mM KH_2_PO_4_, 8 mM Na_2_HPO_4_, pH 7.4) for 20 min and washed three times with PBS. Cell permeabilization was performed by 20 min incubation in 0.1% Triton X-100/PBS followed by 30 min wash in 1% gelatin/PBS (type IV, from bovine skin, Sigma) and 15 min wash in PBS at room temperature (RT). The coverslips were then incubated for 90 min at 37 °C with the specific primary antibody diluted 1:20 in PBS (Tom20: Santa Cruz Biotech., Cat#sc-11415; Parkin: Santa Cruz Biotech., Cat#sc-32282; Drp1: BD Biosciences, Cat#611113; mtHSP60: Abcam, Cat#ab82520; Calreticulin: Thermo Fisher, Cat#PA3-900; Myc: Millipore, Cat#05-724; HA: Cell Signalling, Cat#3724S). Further washing steps with gelatine and PBS were repeated as mentioned before to remove the excess of primary antibody. Staining was revealed by the incubation with specific AlexaFluor secondary antibodies (Thermo Fisher: Goat anti-Rabbit IgG AlexaFluor 405, Cat#A-31556; Goat anti-Rabbit IgG AlexaFluor 594, Cat#A-11012; Donkey anti-Goat IgG AlexaFluor 633, Cat#A-21082; Goat anti-Mouse IgG AlexaFluor 633, Cat#A-21050; Goat anti-Mouse IgG AlexaFluor 488, Cat#A-11001; Goat anti-Rabbit IgG AlexaFluor 488, Cat#A-11008) for 45 min at RT (1:50 dilution in PBS; 1:20 only for Goat anti-Rabbit IgG AlexaFluor 405). After further washing steps, coverslips were mounted using Mowiol 4–88 (Sigma). The coverslips were observed at the SP5 Leica confocal microscope at lasers wavelength of 405, 458, 488, 543, 555, and 633 nm.

### Electron microscopy

Cells were immunogold labeled by the Tokuyasu technique [63]. Briefly, cells were fixed in 2% paraformaldehyde, 0.2% glutaraldehyde in 0.1 M phosphate buffer (PB) for 1 h at RT. Next cells were gently scraped in 1% gelatin and embedded in 12% gelatin in PB. Gelatin squared blocks were infiltrated overnight in 2.3 M sucrose, mounted on aluminum pins, and frozen in liquid nitrogen. Ultrathin cryosections of 70 nm were cut with a Leica FC7 ultramicrotome (Leica Microsystems, Germany), collected on formvar, carbon-coated 150 mesh copper grids and immunolabeled with anti-GFP (Abcam, Cat#ab290) and 10 nm Protein A-gold (Utrecht University, The Netherlands). Grids were then contrasted for 10 min on ice in a solution of 1.8% methylcellusose/0.4% uranyl acetate, air dried on wire loops and observed in a ZEISS Leo912AB (Zeiss, Oberkochen, Germany). Images were acquired using a 2Kx2K bottom mounted slow-scan Proscan camera (Scheuring, Germany) controlled by the EsivisionPro 3.2 (Soft Imaging System, Münster, Germany).

### Image acquisition and processing

Cells were generally imaged 48–72 h after transfection with a Leica TSC SP5 inverted confocal microscope, using either a HCX PL APO 63X/numerical aperture 1.40–0.60 or a HCX PL APO ×100/numerical aperture 1.4 oil-immersion objective. Images were acquired by using the Leica AS software. To count ER–mitochondria contacts, a complete z-stack of the cell was acquired every 0.29 µm. Z-stacks were processed using Fiji [64]: images were first convolved, and then filtered using the Gaussian Blur filter. A 3D reconstruction of the resulting image was obtained using the Volume J plugin (http://bij.isi.uu.nl/vr.htm). A selected face of the 3D rendering was then thresholded and used to count ER–mitochondria contact sites.

### Zebrafish imaging

At 24 hpf, embryos were screened for fluorescence, dechorionated and fixed or anesthetised according to the experiment. To image the co-localization of ER_S_-β_11_ and OMM-GFP_1–10_ with mitochondria and the ER, fish were fixed for 2 h at RT with 4% PFA, then washed with PBS and mounted in low melting agarose (1.3%, Euroclone) on glass coverslips.

For *in vivo* imaging, fish were anesthetised and mounted on 35 × 10 mm glass bottom Petri dishes (Ted Pella, INC. Prod. No. 14023-20) in low melting agarose (1.3%, EuroClone). Fish water containing tricaine methanesulfonate 0.61 mM (Sigma) was added in the Petri dishes, in order to keep fish anesthetised. Mounted fish were imaged at RT (20–23 °C) using a Leica TSC SP5 inverted confocal microscope, using either a HCX PL APO ×63/numerical aperture 1.40–0.60 or a HCX PL APO ×100/numerical aperture 1.4 oil-immersion objective. To count ER-mito contacts, a complete z-stack of the cell was acquired every 0.29 µm. To acquire a representative image of a whole fish expressing pT2-DsRed-UAS-SPLICS_S_-P2A, a ×10 HCPX PL Fluotar NA 0.3 objective was used.

### Ca^2+^ measurements

Ca^2+^ measurements were performed by co-transfecting HeLa cells in a six-well plate with low-affinity mitochondrial aequorin (mtAeqmut) with the two SPLICS moieties in a 1.5:1.5:1 ratio favouring SPLICS. Forty-eight hours post transfection, cells were re-plated into a 96-wells plate (PerkinElmer). mtAeqmut was reconstituted by incubating cells for 1.5 h with 5 µM coelenterazine (Santa Cruz Biotech.) in modified Krebs Ringer Buffer (KRB: 125 mM NaCl, 5 mM KCl, 400 mM KH_2_PO_4_, 1 mM MgSO_4_, 20 mM Hepes, pH 7.4) supplemented with 5 mM glucose at 37 °C. Luminescence measurements were carried out using a PerkinElmer EnVision plate reader equipped with two injector units. After reconstitution, cells were placed in 70 µl of KRB solution and luminescence from each well was measured for 1 min. During the experiment, 100 µM histamine at the final concentration were first injected to activate Ca^2+^ transients, and then a hypotonic, Ca^2+^-rich, digitonin-containing solution was added to discharge the remaining aequorin pool. Output data were analyzed and calibrated with a custom-made macro-enabled Excel workbook.

### Western blot

HeLa cells were seeded in a six-well plate and transfected with three different shRNAs against Mfn2. A scramble shRNA was used as control. At 48 h post transfection, cells were washed with PBS and proteins were extracted for 20 min using ice cold lysis buffer (50 mM Tris-HCl pH 7.4, 150 mM NaCl, 10 mM EGTA, 1% Triton X-100, 1 mM protease inhibitor cocktail (Sigma)). Samples were then centrifuged at 10,000 rpm for 10 min at 4 °C. Protein concentration was determined by the Bradford assay (Bio-Rad). Samples were separated on a 4–15% Mini-PROTEAN TGX™ Precast Protein Gels (Bio-Rad) and blotted using a Immobilon-PSQ PVDF Membrane (Merck Millipore). The membrane was blocked for 1 h at RT using 5% non-fat dried milk in TBST (20 mM Tris-HCl, pH 7.4, 150 mM NaCl, 0.05% Tween-20) and incubated with primary antibodies (Mfn2: Abcam, Cat#ab50838, 1:1000 in TBST, overnight at 4 °C; β-actin: Sigma, Cat#A5441, 1:30,000 in TBST, 2 h at RT; Myc: Millipore, Cat#05-724, 1:1000 in TBST, overnight at 4 °C; HA: Cell Signalling, Cat#3724 S, 1:1000 in TBST, overnight at 4 °C). After three washing steps in TBST, detection was obtained by incubating the membrane with secondary horseradish peroxidase-conjugated antibodies (Santa Cruz Biotech.: Goat anti-Rabbit IgG-HRP, Cat#sc-2004; Goat anti-Mouse IgG-HRP, Cat#sc-2005; 1:2000 in TBST) for 1 h at RT and by incubation with the Luminata HRP substrate (Merck Millipore).

### Statistical analysis

Results shown are mean values ± SEM. Student’s unpaired two-tailed *t-*test was used for comparisons involving two groups when sample followed a Gaussian distribution, otherwise Mann–Whitney test was used. Differences between groups were considered significant when *p* ≤ 0.05. All statistical analyses were performed using GraphPad Prism version 6.00 for Mac OS X, GraphPad Software (La Jolla, California, USA). The exact values of *n* and their means are indicated in the figure legends. *p ≤ 0.05, **p ≤ 0.01, ***p ≤ 0.001, ****p ≤ 0.0001.

## Electronic supplementary material

Supplementary Figures 1-10
