# Supplementary material for: SPLICS: a split green fluorescent protein-based contact site sensor for narrow and wide heterotypic organelle juxtaposition
Source: Cell Death Differ. 2017 Dec 11;25(6):1131–45. doi: 10.1038/s41418-017-0033-z (PMC5988678; doi:10.1038/s41418-017-0033-z)
Supplement: Supplementary file 1 — Supplementary Figures 1-10 [file 41418_2017_33_MOESM1_ESM.pdf]

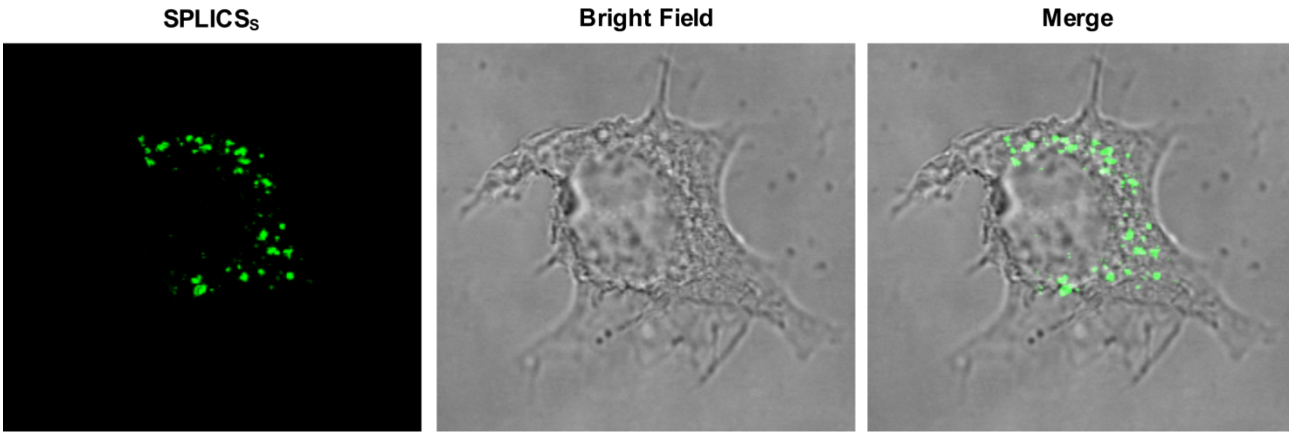

Supplementary Figure S1. HEK293 cells expressing the SPLICS$_S$. HEK293 cells have been transiently transfected with: ER$_s$-β_{11} and OMM GFP$_{1-10}$.

SPLICS$_S$

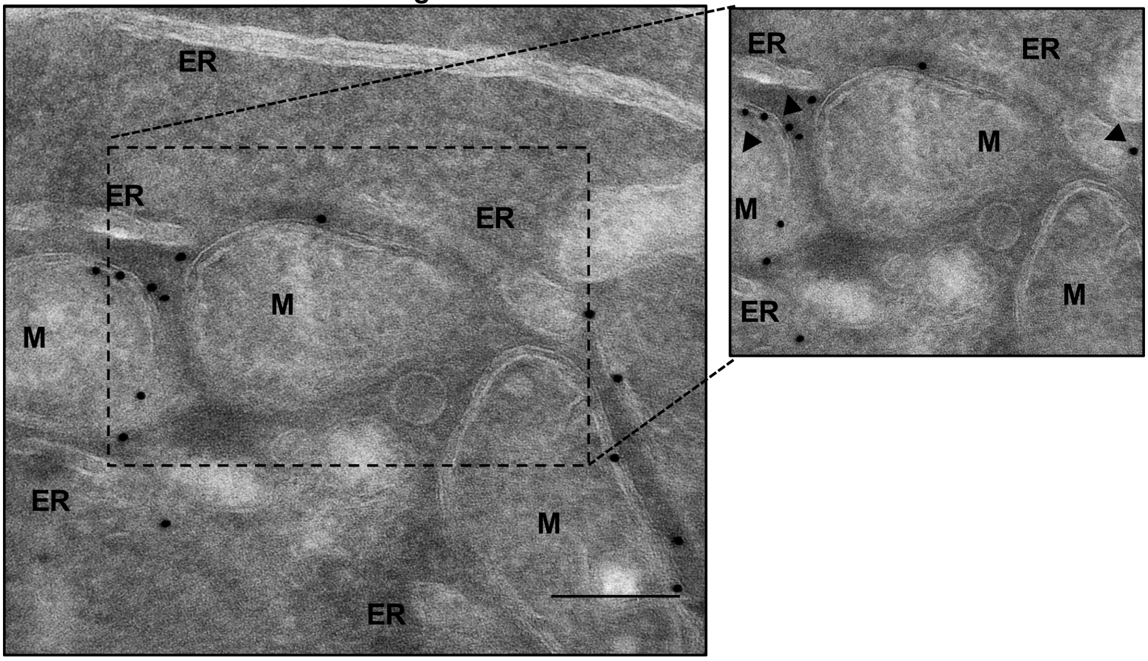

Anti-GFP

Supplementary Figure S2. Immunogold Electron Microscopy images obtained in HeLa cells transiently transfected with the SPLICSS and probed with an anti-GFP polyclonal antibody. Inset is the enlargement of the region defined by the dashed line rectangle. Scale bar: 200 nm.

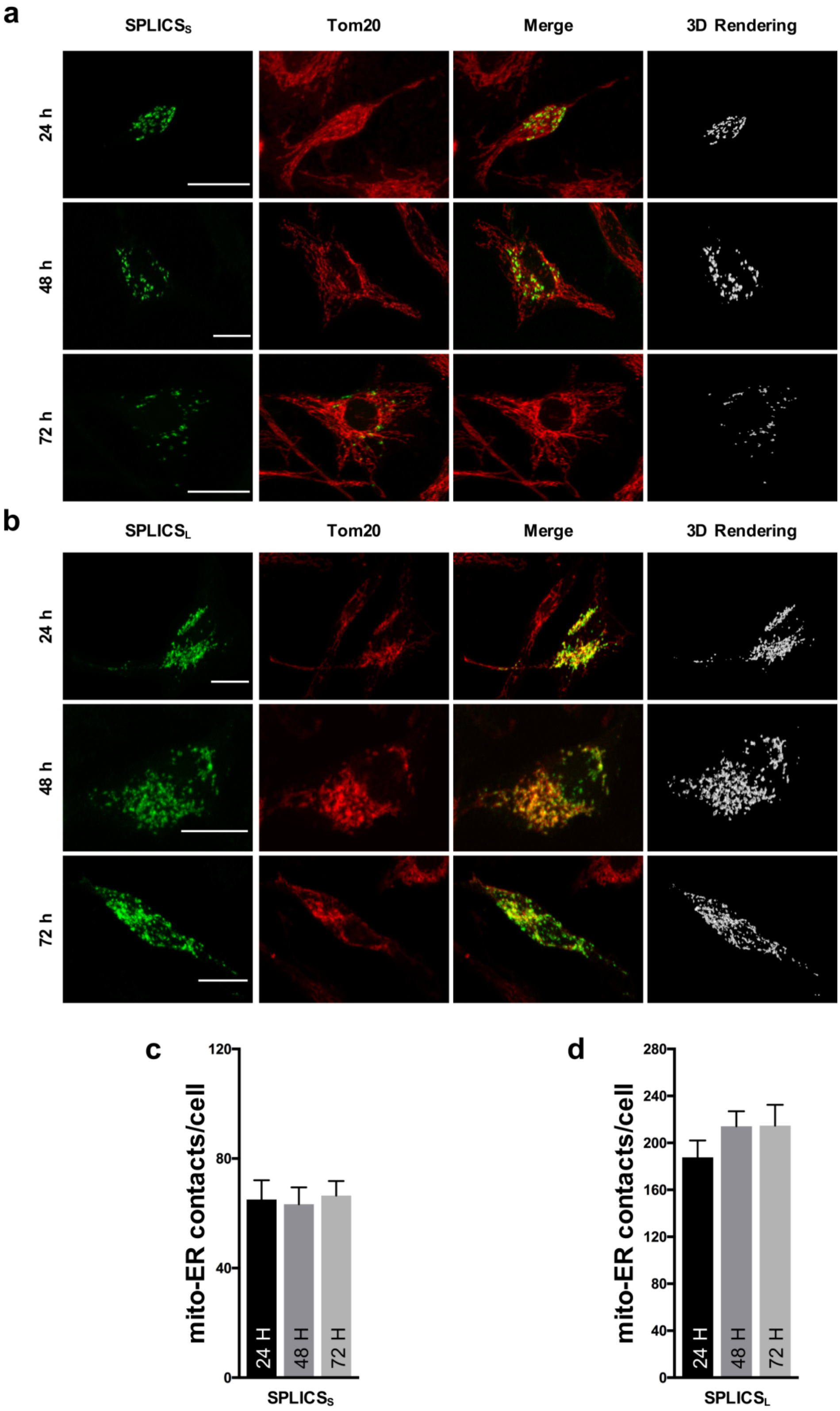

Supplementary Figure S3 Analysis of ER-mitochondria contact sites at different time points. Immunocytochemistry against mitochondria (Tom20, red) is shown in the panels on the middle. The green channel is the merge of several planes. Scale bars, 20 µm. **a**) Representative confocal pictures of HeLa cells expressing the SPLICS$_S$ (upper panels) or SPLICS$_L$ (bottom panels) probe. **b**) Quantification of SPLICS$_S$ contacts by 3D rendering of complete z-stacks. Mean±SEM: 24h 65±7, n=17 cells; 48h 63±6, n=16 cells; 72h 66±5, n=17 cells. **c**) Quantification of SPLICS$_L$ contacts by 3D rendering of complete z-stacks. Mean±SEM: 24h 187±14, n=19 cells; 48h 214±13, n=19 cells; 72h 214±17, n=19 cells.

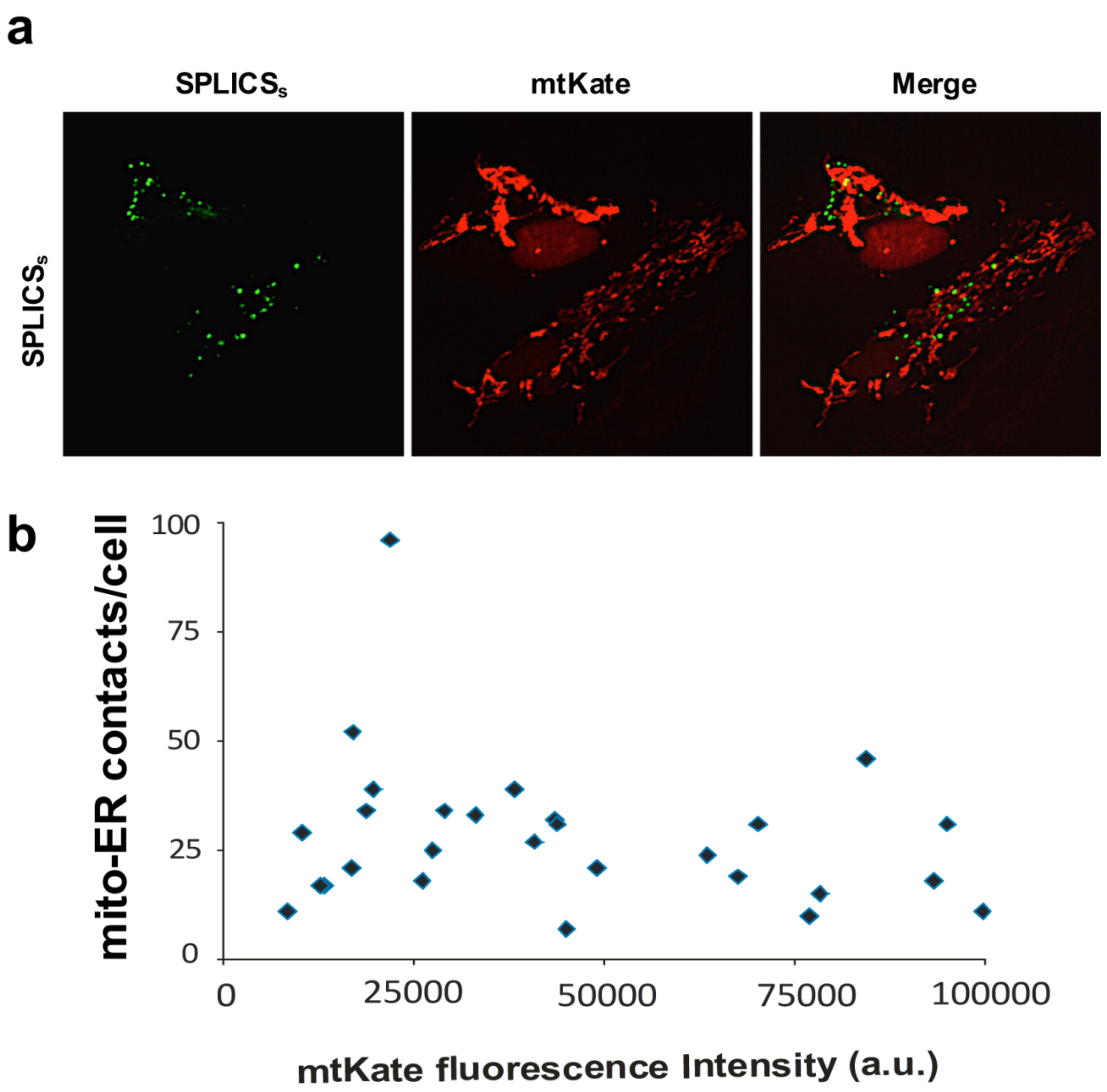

Supplementary Figure S4 The level of protein expression does not affect the amount of ER-mitochondria contact sites present in each cell, thus excluding the possibility of a zippering mechanism. HeLa cells were transiently transfected with mtKate and SPLICS$_S$. Panel a shows two representative cells, both positive for reconstituted SPLICs, with different mtKate expression levels. The graph in panel b reports the relationship between number of contact sites per cell and mtKate fluorescence intensity: the number of contacts does not change accordingly to the increase of the level of probe expression (mirrored by mtKate fluorescence intensity).

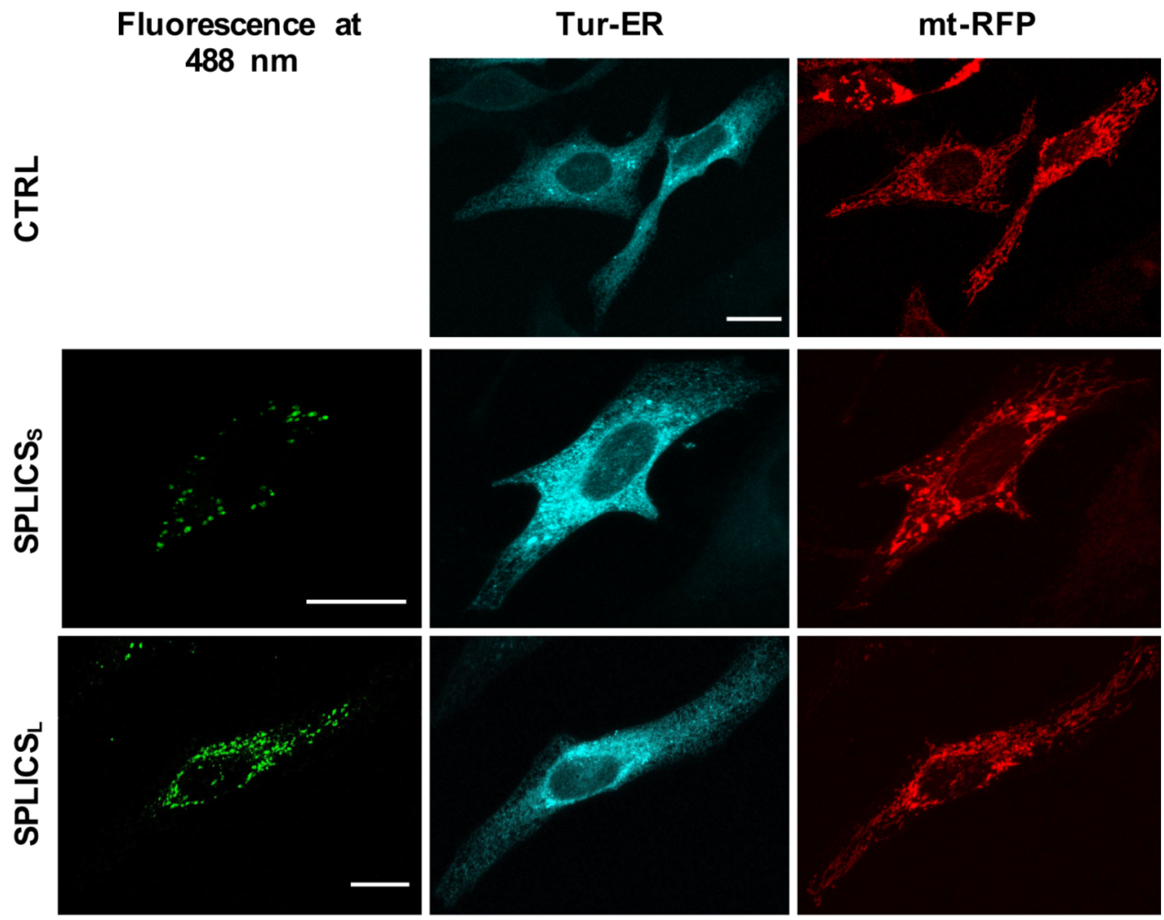

Supplementary Figure S5 Representative images of mitochondrial and ER morphology in HeLa cells expressing the SPLICS probes. pmTurquoise2-ER (Tur-ER) and pTagRFP-mito (mtRFP) were used to label the endoplasmic reticulum and mitochondria, respectively. Scale bars, 20 µm.

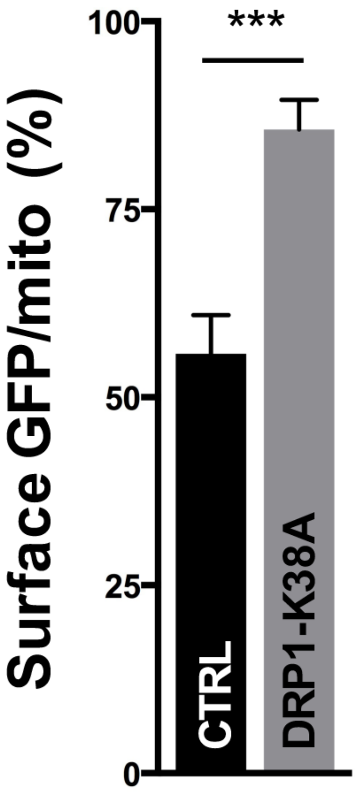

Supplementary Figure S6 Fluorescence area of mitochondria (Tom20) and ER-mitochondria contacts with the $SPLICS_L$ probe. Area was measured from a single plane of each cell. Data shown are the result of 2 independent experiments. Mean±SEM: CTRL 55±5 %, n=18 cells; Drp1-K38A 85±3 %, n=11 cells. *** p≤0.001.

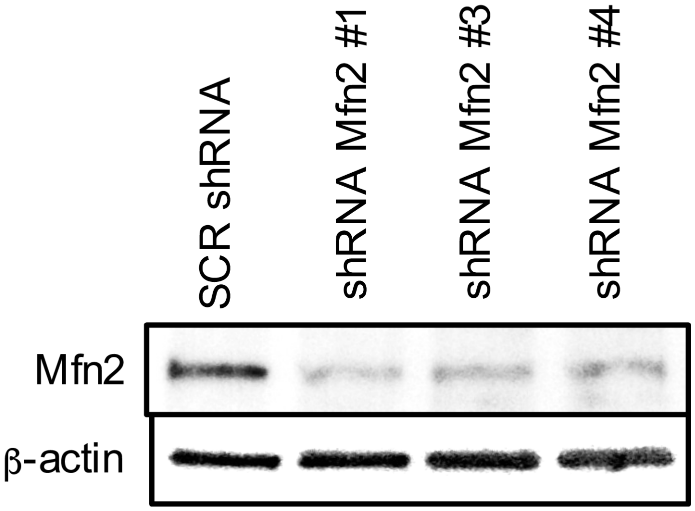

Supplementary Figure S7 Western blot analysis to detect Mfn2 levels, protein lysates from HeLa cells silenced for Mfn2 were loaded in a SDS-page gel and blotted with an anti-Mfn2 antibody. β-actin immunodetection has been used as a loading control.

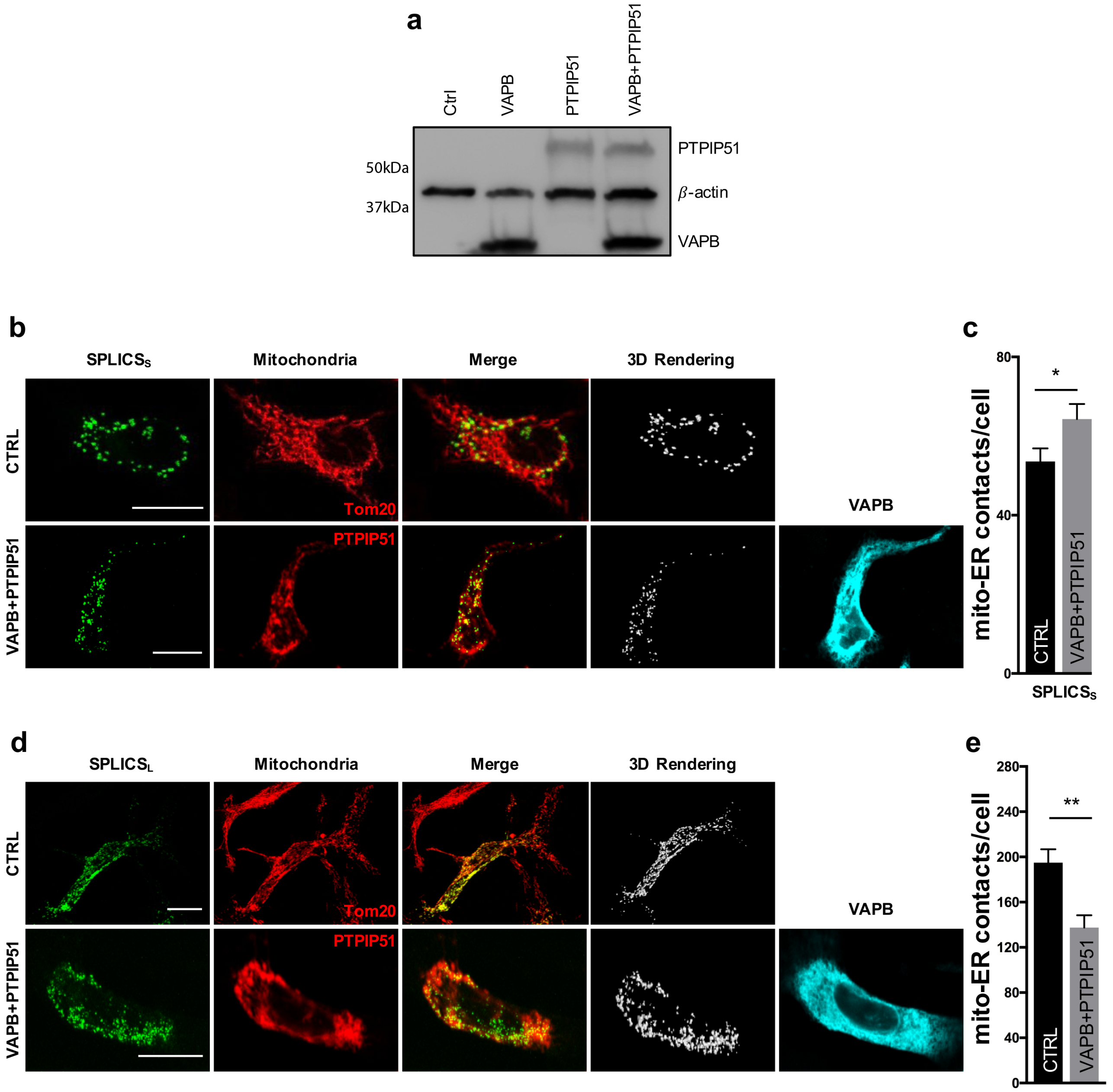

Supplementary Figure S8 Effects of VAPB-PTPIP51 co-expression on ER-mitochondria contacts. Immunocytochemistry analysis against mitochondria (Tom20 or PTPIP51, red) is shown in the panels on the middle. The green channel is the merge of several planes. Scale bars: 20 µm. **a**) Western blot of HeLa cells transfected with void vector or the constructs encoding for VAPB and PTPIP51. **b**) Representative confocal pictures of HeLa cells expressing the $SPLICS_S$ probe. **c**) Quantification of $SPLICS_S$ contacts by 3D rendering of complete z-stacks. Mean±SEM: Ctrl 53±3, n=25 cells; VAPB+PTPIP51 64±3, n=25 cells. **d**) Representative confocal pictures of HeLa cells expressing the $SPLICS_L$ probe. **e**) Quantification of $SPLICS_L$ contacts by 3D rendering of complete z-stacks. Mean±SEM: Ctrl 195±12, n=14 cells; VAPB+PTPIP51 137±11, n=17 cells. *p≤0.05, ** p≤0.01.

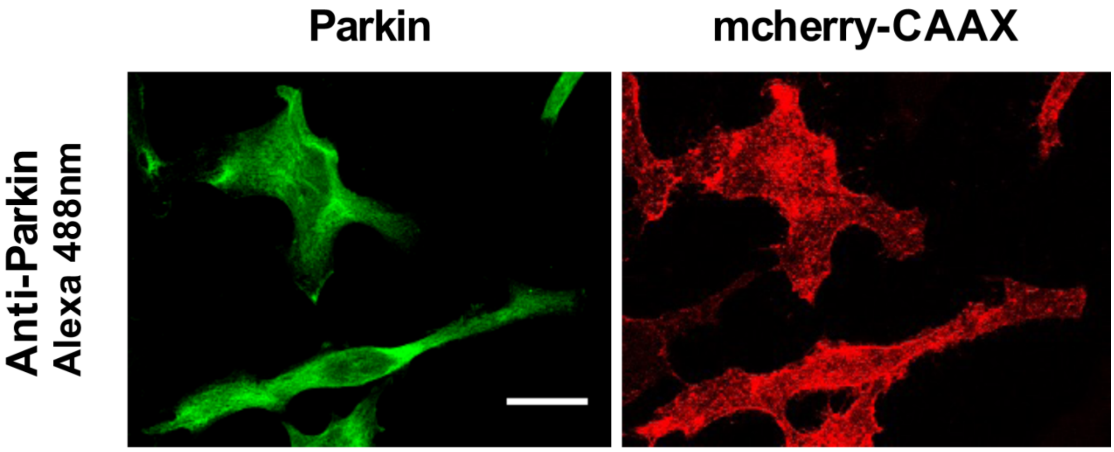

Supplementary Figure S9 Expression of Parkin through a 2A peptide strategy. Parkin coding sequence was cloned upstream of a 2A peptide followed by mcherry-CAAX (plasma membrane). Immunofluorescence against Parkin shows simultaneous expression of both Parkin and mcherry-CAAX in HeLa cells. Scale bar: 20 µm.

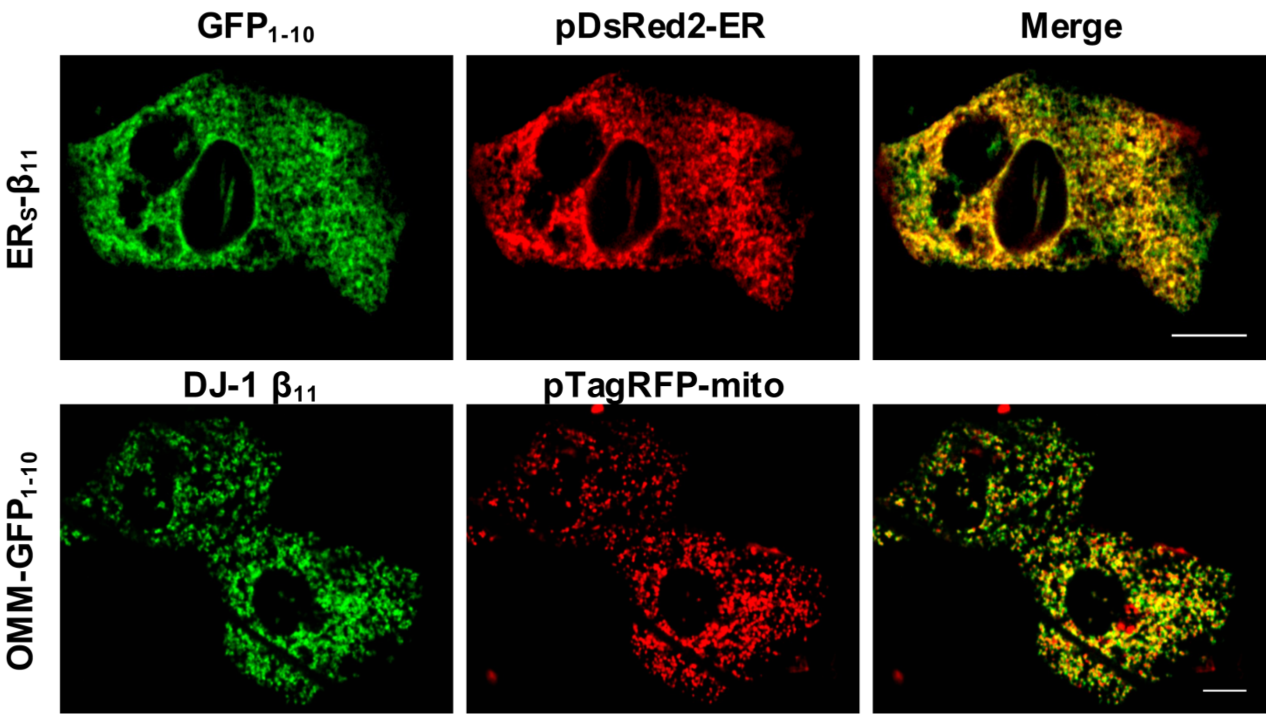

Supplementary Figure S10 Localization of spGFP fragments in zebrafish. The correct targeting of ER_S-β_{11} and OMM-$GFP_{1\text{-}10}$ to ER and mitochondria in zebrafish was verified by complementation with $GFP_{1\text{-}10}$ and DJ-1 β_{11} respectively. To label ER and mitochondria, fertilized eggs were co-injected with pDsRed2-ER or pTagRFP-mito. The pictures showed in this panel have been acquired from a whole embryo transiently expressing the constructs described above. Scale bar: 10 μm.
